# On the Synergism of Biogenic Gold Nanoparticles and Hydroxyaluminum Phthalocyanines in the Photoeradication of *Staphylococcus aureus*

**DOI:** 10.3390/molecules26237378

**Published:** 2021-12-05

**Authors:** Irena Maliszewska

**Affiliations:** Department of Organic and Medicinal Chemistry, Faculty of Chemistry, Wrocław University of Science and Technology, Wybrzeże Wyspiańskiego 27, 50-370 Wrocław, Poland; irena.helena.maliszewska@pwr.edu.pl

**Keywords:** gold nanoparticles, phthalocyanine, phototoxicity, enhancement, photodynamic therapy

## Abstract

Due to the unusual properties of gold nanoparticles, these structures are widely used in medicine and biology. This paper describes for the first time the synthesis of colloidal gold nanoparticles by the cell-free filtrate obtained from the *Coriolus versicolor* biomass and the use of these biogenic nanostructures to increase the photosensitizing efficiency of di- (AlPcS_2_) and tetrasulfonated (AlPcS_4_) hydroxyaluminum phthalocyanines in antibacterial photodynamic therapy. The obtained monodisperse particles were extremely stable, and this remarkable stability was due to the presence of phosphoprotein as a capping agent. The studied gold nanoparticles had a spherical shape, were uniformly distributed, and were characterized by a single plasmon band at wavelength of 514–517 nm. Almost 60% of the gold particles were found to be in the range of 13 to 15 nm. In accordance with the regulations of the American Microbiological Society, indicating that any antimicrobial technique must kill at least 3 log CFU (99.9%) to be accepted as “antimicrobial”, this mortality of *Staphylococcus aureus* was shown to be achieved in the presence of AlPcS_4_ + AuNPs mixture and 4.8 J cm^−2^ light dose compared to AlPcS_4_ alone, which required a light dose of 24 J cm^−2^. The best effect of increasing the effectiveness of combating this pathogen was observed in the case of AlPcS_2_ + AuNPs as a photosensitizing mixture. The light dose of 24 J cm^−2^ caused a lethal effect of the studied coccus in the planktonic culture.

## 1. Introduction

From the time of ancient civilizations, from the Egyptians to the Incas, gold held a special place of real and symbolic value to mankind [1]. Gold was considered the metal of the gods derived from the sun, and was therefore widely used in ancient Rome to make various ornaments, ceramics, and tapestries. Gold was used as money exchange, as an investment, and as a jewelry.

The interest in colloidal gold occurred somewhat later, and dates back to the fourth century of the Lycurgus cup. This cup is made of glass that changes color depending on the location of the light source. Today it is obvious that this cup owes its uniqueness to colloidal gold and silver [2]. However, the groundbreaking interest in colloidal gold did not begin until the mid-1850s. Michael Faraday noticed that the reduction in gold chloride leads to a “ruby” solution that scatters light [3]. After Faraday’s classic work on the preparation of gold colloids, it became clear that the bright red colors of ancient paints and stained glass were caused by presence of this element in a nano-sized form. Among the various metallic nanoparticles, gold nanoparticles are believed to be the most stable and biocompatible, and can now be prepared in a variety of shapes, including nanospheres, nanorods, nanocubes, nanowires, nanobipyramids, nanoflowers, nanocoatings, nanowires, and nanocages [4,5]. These days, it is well known that the unique properties of gold colloids depend on the size and shape, high surface to volume ratio, and surface area that can be easily modified with ligands containing functional groups, such as thiols, phosphines, and amines [6]. Due to the unusual properties of gold nanoparticles, these structures have found wide applications in medicine, biology, food industry, and water treatment [7].

One of the important medical techniques developed in recent decades is photodynamic therapy (PDT)—a method of photo-destroying various pathogenic cells. PDT is an effective light therapy based on the combined action of three main components: a non-toxic photosensitizer (PS), and light and molecular oxygen (O_2_). Under appropriate light irradiation, the photosensitizer is activated, being able to absorb and transfer electrons, while oxygen molecules present in situ act as electron acceptors [8,9]. Therefore, cytotoxic reactive oxygen species (ROS) are generated. These reactive oxygen species (oxidizing radicals and singlet oxygen), react with neighboring biological macromolecules causing significant cytotoxicity and inducing the death of pathogenic cells. Singlet oxygen is usually considered to be the most reactive species responsible for lethal cell damage [10]. Typical molecules oxidized include amino acid residues in proteins (cysteinyl, histidyl, methionyl, tryptophyl, and tyrosyl), guanine residues in nucleic acids, unsaturated lipids/phospholipids, and cholesterol.

The literature data have shown that gold nanoparticles are most often considered in terms of their use in photodynamic therapy of cancer cells. Initially, colloidal gold was applied as a simple photosensitizer carrier [11], but in recent years this technique has evolved considerably and covers such issues as: the use of a PEGylated modification for water compatibility [12], singlet oxygen production enhanced by gold surface [13], surface functionalization of nanoparticles with biological ligands to specifically target overexpressed receptors on the surface of pathogenic cells [14], the synthesis of nanorods and nanostars to enable combined PDT and photothermal therapies [15]. These versatile applications of gold nanoparticles have significantly increased the effectiveness of traditional photosensitizers in cancer therapy both in vitro and in vivo. Moreover, colloidal gold has also found interest in the photodynamic inactivation of pathogenic microbes (aPDI). For instance, Singh at al. [16] described antimicrobial activity of gold nanoparticles against *E. coli* by absorbing light and converting it into heat. An inhibition of the growth of *C. albicans* by thermal therapy with gold nanoparticles was also reported by Wani and Ahmad [17], and Yu et al. [18].

Our previous studies concerned the use of gold nanoparticles to enhance photodestruction of bacterial cells with Methylene Blue (MB) as a photosensitizer [19]. It has been proven that biogenic gold nanoparticles prevent rapid photobleaching of MB, thus enhancing the photoactivity of this dye, which results in an excellent bactericidal effect.

This paper describes for the first time the synthesis of monodisperse and very stable gold nanoparticles by the cell-free filtrate of *Coriolus versicolor,* and the synergistic effect between these biogenic gold nanoparticles and sodium salt of di- (AlPcS_2_) and tetrasulfonated (AlPcS_4_) hydroxyaluminum phthalocyanines in the photoeradication of *S. aureus.* The possible mechanism of this phenomenon is discussed.

## 2. Results

### 2.1. Formation and Characterization of the Biogenic Gold Nanoparticles

**Figure 1:**
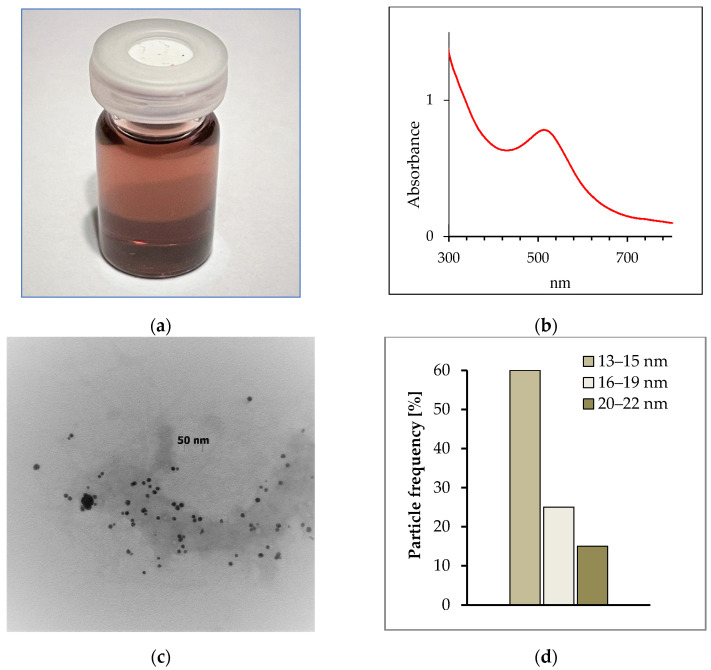
The color of the cell-free filtrate of *Coriolus versicolor* turning red after incubation with the gold ions: (**a**) absorption spectrum of the biogenic gold nanoparticles; (**b**) TEM image of the gold nanoparticles synthesized by the cell-free filtrate; (**c**) histogram of the size distribution of the gold nanoparticles (**d**).

The procedure for the synthesis of colloidal gold by a cell-free filtrate obtained from the *Coriolus versicolor* biomass was described in the Supplementary Materials. The formation of gold nanoparticles by the cell-free filtrate was visually monitored by changing the color of the filtrate from light yellow containing the gold ions to bright red at the end of the second day of incubation, suggesting colloidal gold synthesis (Figure 1a). In contrast, the color of the filtrate without the addition of the gold ions remained unchanged throughout the incubation time. The presence of gold nanoparticles in the filtrate after incubation with the gold ions was confirmed by UV–Vis spectroscopy (Figure 1b). As can be seen, this spectrum shows an intense peak at 514–517 nm corresponding to the resonance frequency of the surface plasmons of gold nanocrystalline particles [20]. A representative TEM image of the resulting gold nanoparticles is presented in Figure 1c. This image indicates that the gold nanoparticles are spherical and relatively uniform in diameter. The particle size histogram (Figure 1d) shows that the sizes of synthesized gold nanostructures ranged from 13 to 22 nm and it is estimated an average size of 14 ± 3 nm. The size distribution presents in the histogram reveals that nearly 60% of the gold particles fall in the size range of 13 to 15 nm (Figure 1d). It should be emphasized that the particle size obtained from dynamic light scattering measurements was larger than that estimated from TEM measurements, and amounts to 22 ± 3 nm. The zeta potential of this colloidal gold showed the value of −18 ± 3 mV, which indicated the great stability of the particles in the aqueous suspension.

**Figure 2:**
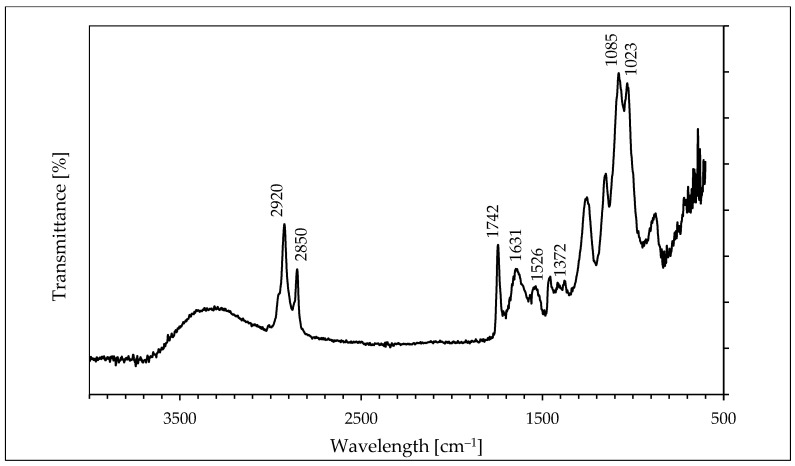
FTIR spectrum of the gold nanoparticles synthesized by the cell-free filtrate of *Coriolus versicolor*.

FTIR measurements (Figure 2) were performed to identify possible interactions between gold ions and fungal molecules that act as a reducing and capping agent in the synthesis and stabilization of gold nanoparticles. The broad band appearing in the range of 3000–3450 cm^− 1^ is the summation of associative intermolecular hydrogen bonds arising from -NH_2_ and -OH groups. The bands noticed at 2850 cm^−1^ and 2920 cm^−1^ assigned to the symmetric and asymmetric stretching vibration of sp^3^ hybridized CH_2_ groups respectively. It is worth noting that the band at ~1742 cm^−1^ (corresponds to the vibration of carbonyl stretching in ketones, aldehydes, and carboxylic acids) suggests that the reduction in the gold ions can be coupled to the oxidation of hydroxyl groups. The spectrum of colloidal gold exhibits intense bands at 1631 cm^−1^ and 1526 cm^−1^ corresponding to the amide I and II bands of proteins, respectively. The two bands observed at 1372 cm^−1^ and 1023 cm^−1^ can be attributed to C–N stretching vibrations of aromatic and aliphatic amines, respectively.

The peaks at 1085 cm^−1^ and 1024 cm^−1^ correspond to P−OH and P−O−C stretching, respectively, indicating the presence of protein phosphate groups. The position of these bands is close to those reported for native proteins [21]. It should be emphasized that detailed interpretation in the fingerprint region (~1300 to 900 cm^−1^) is difficult due to the extensive overlapping of the amide-III band with in-plane deformational modes of O−H bond (1450−1250 cm^−1^) as well as C−O vibrations of esters (1330−1050 cm^−1^) and of aromatic anhydrides (1282−1220 cm^−1^).

### 2.2. Cytotoxicity Studies

The first set of our experiments was examined the darkness cytotoxicity of AlPcS_2_, AlPcS_4_, and the biogenic gold nanoparticles. As can be seen in Table S1 (Supplementary Materials), the studied phthalocyanines at concentrations of 7 and 14 mgL^−1^ were non-toxic to the studied coccus. Changes in the value of CFU mL^−1^ were within the measurement error. When AlPcS_2_ and AlPcS_4_ were used at a concentration of 28 mgL^−1^, the viable count of *S. aureus* was reduced by 39 ± 1.5% and 22.4 ± 1.5%, respectively (*p* < 0.05). After 30 min of incubation of the gold nanoparticles with *S. aureus*, a slight reduction of 32.4 ± 1.0% in the number of viable cells was obtained. The possibility of endogenous activation of photosensitizers by the influence of laser light alone on the viability of *S. aureus* was also investigated. It was shown that exposure of the studied bacteria to laser light for up to 30 min (energy fluence of 144 J cm^−2^) did not result in a significant bacterial killing (mortality did not exceed 45%) (*p* < 0.05) (Table S2; Supplementary Materials).

### 2.3. The Effectiveness of aPDI with AlPcS_2_ and AlPcS_4_ as Photosensitizers

**Figure 3:**
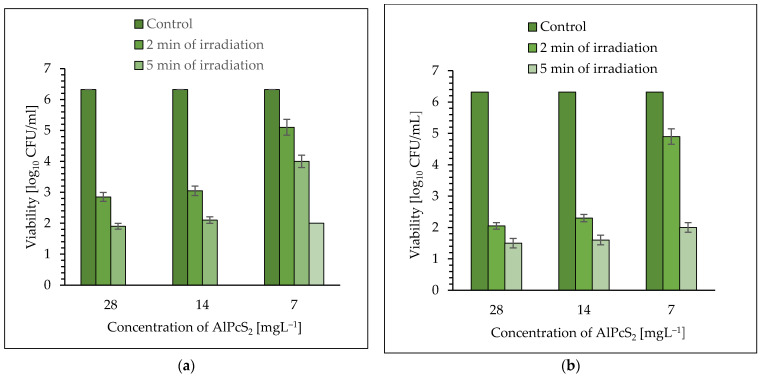
The effect of AlPcS_2_ on viability of *S. aureus* after exposure to laser light for 2, 5, and 10 min. (**a**) laser light (λ = 650 nm; the radiation power density of 105 mW cm^−2^); (**b**) laser light (λ = 650 nm; the radiation power density of 210 mW cm^−2^ ). Average SD of three independent experiments is shown. The efficiency of this treatment was considered as statistically significant for all groups (*p* < 0.05).

As can be seen in Figure 3a,b, the efficiency of photo-killing of *S. aureus* depends on the concentration of photosensitizer and dose of laser light. It was observed that an excellent reduction in *S. aureus* viability was found when AlPcS_2_ was used as a photosensitizer. This dye at a concentration of 28 mgL^−1^ showed 3.41 log_10_ and 4.42 log_10_ unit reduction in bacterial cells after 2 and 5 min of irradiation with laser light of 650 nm (Figure 3a) (*p* < 0.05). The significant kill of viable pathogenic cells was achieved after 2 and 5 min of irradiation in the presence of AlPcS_2_ at a concentration of 14 mgL^−1^ and this was a cfu reduction of 3.27 log_10_ and 4.22 log_10_ units, respectively (*p* < 0.05). It was observed that a ten-minute treatment of pathogenic cells in the presence of AlPcS_2_ at the concentrations of 28 and 14 mgL^−1^, resulted in a lethal effect (the number of bacteria was below the detection level).

Time-dependent mortality of culture was also found after 2, 5, and 10 min of exposure of cells to laser light using AlPcS_2_ at a concentration of 7 mgL^−1^ and it was estimated that these treatments resulted in a reduction in cfu of 1.22 log_10_, 2.32 log_10_, and 4.32 log_10_, respectively (*p* < 0.05) (Figure 3a).

Appreciable eradication of *S. aureus* cells was achieved using laser light with a radiation power density of 210 mW cm^−2^. The AlPcS_2_ at a concentration of 28 mgL^−1^ showed 4.27 log_10_ and 4.82 log_10_ unit reduction in the bacterial planktonic cells after 2 and 5 min of irradiation, respectively (*p* < 0.05) (Figure 3b). It was found that after 2 and 5 min of light treatment, in the presence of AlPcS_2_ at a concentration of 14 mgL^−1^, the viable count showed a reduction of 4.02 log_10_ and 4.72 log_10_ unit, respectively (*p* < 0.05). It is worth emphasizing that AlPcS_2_ at a concentration of 7 mgL^−1^ showed a high photo-sensitizing activity and after 2 and 5 min of irradiation the reduction in the number of viable cells was 1.42 log_10_ and 4.32 log_10_ unit, respectively (*p* < 0.05). Ten-minute exposure of the pathogenic bacteria to laser light with a radiation power density of 210 mW cm^−2^, regardless of the concentration of the photosensitizer (AlPcS_2_), resulted in a lethal effect (the number of bacteria was below the detection limit).

**Figure 4:**
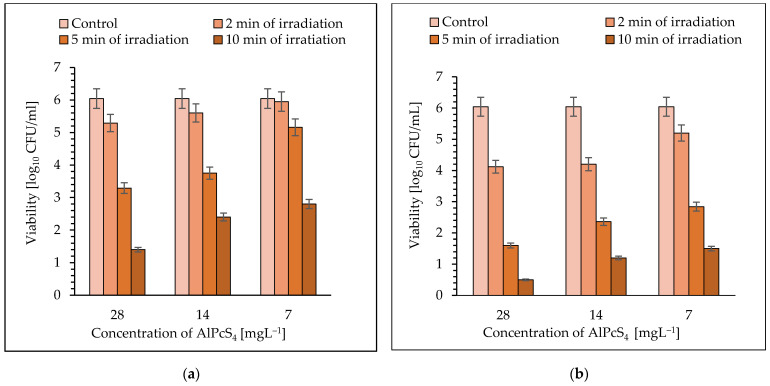
The effect of AlPcS_4_ on viability of *S. aureus* after exposure to laser light for 2, 5, and 10 min. (**a**) laser light (λ = 650 nm; the radiation power density of 105 mW cm^−2^); (**b**) laser light (λ = 650 nm; the radiation power density of 210 mW cm^−2^). Average SD of three independent experiments is shown. The efficiency of this treatment was considered as statistically significant for all groups (*p* < 0.05).

Irradiation of *S. aureus* for 2, 5, and 10 min with laser light (power density of 105 mW cm^−2^) in the presence of AlPcS_4_ at a concentration of 28 mgL^−1^, resulted in a reduction in cfu by 0.75 log_10_, 2.75 log_10_, and 4.64 log_10_ unit, respectively (*p* < 0.05) (Figure 4a). The significant reduction in viable count for AlPcS_4_ at a concentration of 14 mgL^−1^ was achieved after 2, 5, and 10 min of red-light exposure and it was 0.44 log_10_, 2.29 log_10_, and 3.64 log_10_, respectively (*p* < 0.05). When the AlPcS_4_ at a concentration of 7 mgL^−1^ was used in the experiment, the reduction in viability of bacterial cells was 0.84 log_10_, 3.2 log_10_, and 4.9 log_10_ unit, after 2, 5, and 10 min of irradiation, respectively (*p* < 0.05). A 30-min exposure of *S. aureus* to laser light with radiation power density of 105 m Wcm^−2^, regardless of the concentration of AlPcS_4_, resulted in lethal effect (the number of bacteria was below the detection level) (Figure 4a).

When a laser with radiation power density of 210 m Wcm^−2^ and the AlPcS_4_ at a concentration of 28 mgL^−1^ was used in the experiment, a reduction in the number of viable cells by 1.92 log_10_ and 4.44 log_10_ units after 2 and 5 min of irradiation, respectively, was found (Figure 4b) (*p* < 0.05). A longer exposure time (10 min) increased in cell mortality up to 5.54 log_10_ unit. When the AlPcS_4_ at a concentration of 14 mgL^−1^ was applied as a photosensitizer, a reduction in viability of the coccus was 1.84 log_10_, 3.68 log_10_, and 4.84 log_10_ unit, after 2, 5, and 10 min of irradiation, respectively (*p* < 0.05). It was found that after 2, 5, and 10 min of light treatment, in the presence of AlPcS_4_ at a concentration of 7 mgL^−1^, the viable count showed a reduction of 0.84 log_10_, 3.2 log_10_, and 4.54 log_10_ unit, respectively (*p* < 0.05) (Figure 4b).

In the case of laser light with a power density of 210 mW, regardless of the concentration of AlPcS_4_, the lethal exposure of bacteria to this light was 30 min.

### 2.4. The Effect of Gold Nanoparticles on the Effectiveness of aPDI

**Figure 5:**
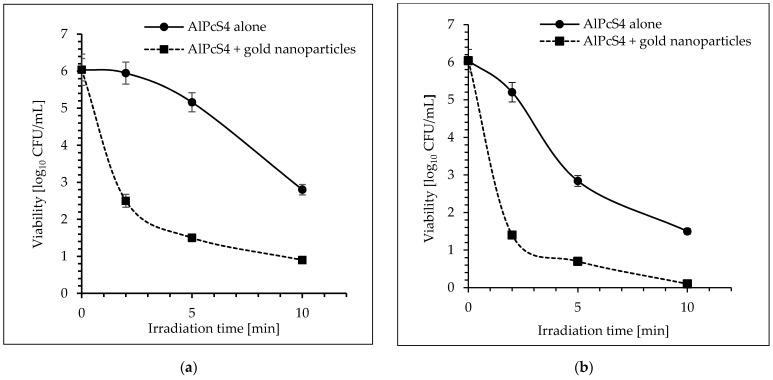
The effect of gold nanoparticles on the effectiveness of photo-killing of *S. aureus* with AlPcS_4_ as a photosensitizer. (**a**) laser light (λ = 650 nm; the radiation power density of 105 mW cm^−2^); (**b**) laser light (λ = 650 nm; the radiation power density of 210 mW cm^−2^). Average SD of three independent experiments is shown. The efficiency of this treatment was considered as statistically significant for all groups (*p* < 0.05).

A greatly enhanced photokilling of *S. aureus* were obtained when the studied cocci were irradiated with laser light in the presence of mixture of AlPcS_4_ at a concentration of 7 mgL^−1^ and the gold nanoparticles at a concentration of 20 ppm (Figure 5a,b). As can be seen in Figure 5a, after just two minutes of exposure of bacteria to laser light, a significant reduction in the number of viable cells by 3.54 log_10_ units was observed in the presence of AlPcS_4_ + AuNPs mixture (*p* < 0.05). Longer treatment time (5 and 10 min) resulted in 4.54 log_10_ and 5.14 log_10_ units of viability reduction, respectively (*p* < 0.05). Extending the treatment time to 30 min resulted in a lethal effect. The similar effective eradication was observed using laser light with a radiation power density of 210 m Wcm^−2^. The mixture of AlPcS_4_ + AuNPs showed 4.64 log_10_ unit reduction in bacterial planktonic cells after 2 min of irradiation (*p* < 0.05) (Figure 5b). The longer irradiation times for pathogenic cells of 5 and 10 min resulted in a reduction in viability of 5.34 log_10_ and 5.94 log_10_ units, respectively (*p* < 0.05). The 30-min light treatment was lethal to the bacterial cells.

**Figure 6:**
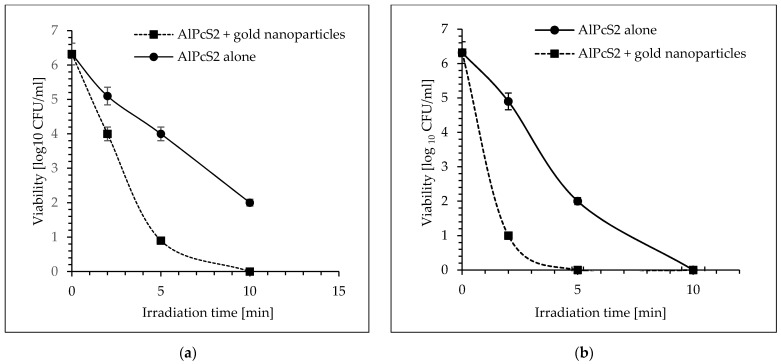
The effect of gold nanoparticles on the effectiveness of photokilling of *S. aureus* with AlPcS_2_ as a photosensitizer. (**a**) laser light (λ = 650 nm; the radiation power density of 105 mW cm^−2^); (**b**) laser light (λ = 650 nm; the radiation power density of 210 mW cm^−2^). Average SD of three independent experiments is shown. The efficiency of this treatment was considered as statistically significant for all groups (*p* < 0.05).

As can be seen in Figure 6a, a mixture of AlPcS_2_ + AuNPs enhanced photoeradication of *S. aureus* as 2 and 5 min of irradiation (power density of 105 mW cm^−2^) resulted in 2.32 log_10_ and 5.42 log_10_ units of viability reduction, respectively (*p* < 0.05). The 10-min laser light treatment induced a lethal effect on the *S. aureus* cells.

The similar bactericidal effect was also observed using laser light with a radiation power density of 210 mW cm^−2^. The mixture of AlPcS_2_ and gold nanoparticles showed 5.32 log_10_ unit reduction in the bacterial planktonic cells after 2 min of irradiation (*p* < 0.05) (Figure 6b). Extending the irradiation time of the studied coccus to 5 min resulted in lethal effect (the number of bacteria was below the detection limit) (*p* < 0.05).

## 3. Discussion

This paper describes a simple biological method for the production of monodisperse and stable colloidal gold nanoparticles (AuNPs), and the use of these nanostructures to increase the photobactericidal activity of sulfonated hydroxyaluminum phthalocyanines against *S. aureus*.

The colloidal gold nanoparticles were synthesized by the cell-free filtrate obtained from biomass of *Coriolus versicolor*, and this protocol was presented for the first time. My findings strongly supported the role of cell-free filtrate of C. *versicolor* as a reducing and stabilizing factor, which is in agreement with a number of previous reports [21,22,23]. The metabolic ability of *Coriolus versicolor* to synthesize gold nanoparticles was previously described by Sangha and Verma [21], but the authors used the biomass of this organism to prepare gold nanostructures. The authors speculated that gold nanoparticles were formed on the fungal cell walls, and this biosynthesis involved several steps. First, the gold ions were trapped and reduced by the proteins on the cell surface at a pH ranging from 2.0 to 3.5, forming nuclei, followed by extensive crystal growth into the final shapes and size. The positive amino and sulfhydryl groups of proteins on the mycelial surface, made the gold ions available for binding and allowed the reduction of Au(III) to Au(0). The participation of carboxyl groups (abundant in biomass and protonated at low pH) in the binding of Au(III) ions was also not excluded.

The biogenic nanostructures used in this study were subsequently characterized by a number of techniques including UV–Vis, transmission electron microscopy (TEM), DLS technique, and Fourier transform infrared spectroscopy (FTIR). The findings strongly supported the role of the cell-free filtrate of *C. versicolor* as a reducing and stabilizing factor, which is in agreement with some previous reports [20]. The obtained monodispersed nanoparticles are stabilized by capping agent, which is likely to be surface-bound phosphoprotein [22]. The presence of protein as coating agent determines the extremely good stability of these nanoparticles, and their aggregation was not observed even after 15 months of storage.

The TEM image showed that the colloidal gold had spherical shapes and was relatively uniform. It is noteworthy that the size of these particles determined by the DLS method was about 60% larger than that estimated by TEM microscopy. This phenomenon is related to the fact that the overall size of the biogenic nanoparticles is greatly increased due to the presence of hydrated coating agents (possibly proteins) as well as solvation effects [24].

The studied antibacterial photodynamic therapy enhanced by AuNPs is one of the important bactericidal techniques developed in the last decade due to its high efficiency and, unlike conventional antibiotics, rarely causing bacterial resistance [24].

Di- and tetrasulfonated hydroxyaluminum phthalocyanines used in our experiments are water soluble molecules characterized by the lack of toxicity to normal tissues, a high quantum yield of triplet production, a high efficiency of production of active forms of oxygen, and a sufficient light stability [25]. It was found that the lethal effect against *S. aureus* was achieved in the presence of AlPcS_2_ and AlPcS_4_ (regardless of the concentration of these photosensitizers) at the light doses of 24 J cm^−2^ and 72 J cm^−2^, respectively. From these results, it was concluded that the number of sulfate groups in the structure of the studied dyes influenced their photo-bactericidal activity, because the effectiveness of aPDT in the presence of the AlPcS_2_ was higher than that of the AlPcS_4_. These findings are consistent with previous observations of some authors who explained that the degree of sulfonation is inversely correlated with the lipid/water partition coefficient of phthalocyanine [26,27,28], and the reduction in the number of sulfonate groups increases the lipophilicity of the dye. It is believed that the reduction in the membrane permeability barrier for lipophilic molecules results in a faster and higher uptake of the less sulfonated phthalocyanine than the more sulfonated derivative [26]. Taking into account our previous results that AlPcS_4_ up to a concentration of 10 µg/mL is non-toxic to normal human keratinocytes (KERTr) and normal endothelial cells (HUVEC) [29], an attempt was made to increase the photo-bactericidal effect of di- and tetrasulfonated hydroxyaluminum phthalocyanines at a concentration of 7 µg/mL. Therefore, in the next set of our experiments, mixtures of hydroxyaluminum phthalocyanines and biogenic nanoparticles were used as photosensitizers.

It was found that in the presence of AlPcS_4_ + AuNPs mixture, a lethal effect of *S. aureus* was achieved with energy fluence of 72 J cm^−2^ (Table 1). From these results it can be concluded that the presence of gold nanoparticles in the photosensitizing mixture did not increase the effectiveness of the photodynamic therapy, because a similar dose of light was required to obtain a lethal effect using AlPcS_4_ alone. Taking into account the regulations of the American Microbiological Society, indicating that any antimicrobial technique must kill at least 3 log CFU (99.9%) to be accepted as “antimicrobial”, it should be emphasized that such a mortality rate of the tested bacteria was achieved in the presence of AlPcS_4_ + AuNPs mixture and a light dose of 4.8 J cm^−2^ compared to AlPcS_4_ alone, which required a light dose of 24 J cm^−2^. An excellent effect of enhancing the effectiveness of photodynamic therapy was observed in the case of AlPcS_2_ + AuNPs as a photosensitizing mixture. The light dose of 24 J cm^−2^ caused a lethal effect.

An understanding the role of the biogenic gold nanoparticles in increasing the effectiveness of aPDT remains an open question at this stage of our studies. Previously, some researchers found that gold nanoparticles can enhance the efficacy of photosensitizers such as phthalocyanines, toluidine blue O, indocyanine green, and hematoporphyrin [30]. It is assumed that by placing the photosensitizer (PS) near the gold nanoparticle (about 10 nm from the metal surface), the PS electrons involved in the excitation/emission process interact with the plasmonic field of metal nanoparticles [31]. This interaction results in a quenching or an increase in the level of PS fluorescence and consequently in the concentration of radicals. Khaing Oo et al. [32] showed that the conjugation of protoforphyrin IX on the surface of gold nanoparticles increases the production of ROS in a size dependent manner. Theoretical simulations of the electromagnetic field amplification by gold nanoparticles confirmed that ROS production is significantly enhanced by the localized plasmonic field of gold nanoparticles. Although several reports have shown that increased ROS production is possible by chemical coupling of a monomeric photosensitizer on the surface of gold nanoparticles, it has been suggested that covalent bonding of PS to the nanostructure matrix is not necessary to obtain photodynamic enhancement.

For example, Yang et al. [33] showed that ROS production by free protoporphyrin IX can be enhanced twice in the presence of gold nanoparticle aggregates. This phenomenon is explained by the fact that when metallic nanoparticles are close, their transition dipoles connect with each other and the intensity of the localized electromagnetic field can be distributed, creating regions of a highly confined electric field. Due to the coherent interference of the amplified fields at the particle junction, larger electromagnetic fields are created, which in turn enhance the energy supplied to the photosensitizer. Importantly, due to the high absorbance of gold nanoparticles aggregated in the NIR region, increased ROS production was observed even after excitation by longer wavelengths (λ > 600 and λ > 700 nm) [34]. Narband et al. [35] prepared a photosensitizing system by covalent bond between toluidine blue and gold nanoparticles. This photosensitizer showed increased photoactivity against *S. aureus* but significantly less ^1^O_2_ was produced. The authors suggested that the adsorption of TBO molecules on the surface of gold nanoparticles allowed the collection of more light falling on the molecule, as evidenced by an increase in the extinction coefficient of adsorbed TBO. This created a PS excitation state that decayed quickly due to some dark process. Nevertheless, the non-radiation process did not allow ^1^O_2_ to be produced. Presumably, TBO was suppressed by the redox pathway, resulting in the production of other ROS, possibly hydroxyl radicals, which may explain the increased antibacterial photoactivity.

Our results cannot be compared with those obtained by other authors because the conditions of the experiment were significantly different. For example, Mantareva et al. [36] showed that aPDI results in intensive killing of *S. aureus* treated with cationic ZnPcMe and anionic ZnPcS, at irradiance of 100 mW cm^−2^ and fluence rate of 60 J cm^−2^.

In subsequent experiments, attempts were made to explain the mechanism of enhancing the photosensitization of bacteria by the biogenic gold nanoparticles. Initially, the preventive effect of nanoparticles on the photobleaching of sulfonated hydroxyaluminum phthalocyanines was investigated, but no significant difference was observed in this process in the presence of AuNPs.

Secondly, according to the procedure described in [33], the production of ROS in the presence of gold nanoparticles was estimated and also no significant differences were found. The influence of gold nanoparticles on the transport of AlPcS_2_ and AlPcS_4_ to *S. aureus* cells was also studied (see Supplementary Materials), but no significant differences in the number of dyes taken up by the cells were detected. The observed phenomenon of the enhancement of the photobactericidal effect of hydroxyaluminum phthalocyanines by the biogenic gold nanoparticles synthesized by the cell-free filtrate of *Coriolus versicolor* remains a problem to be solved in further works.

It is worth remembering that one of the side effects of photodynamic therapy is pain. So far, only a few studies have looked at the correlation between the light dose used in PDT and the level of induced pain. For example, Erison et al. [37] described that PDT in actinic keratosis should be performed with a total light dose of 40 J cm^− 2^ using inconsistent light sources, and this treatment results in little pain (most patients rate the pain as ’three’). Attili et al. [38] showed that the energy fluence of 75 J cm^−2^ caused moderate pain (most patients rated the pain as ’six’) in phototherapy for non-melanoma skin cancer. The presented results showed that the use of the AlPcS_2_ + AuNPs mixture allowed to achieve the lethal effect of *S. aureus* at an extremely low light dose of 4.8 J cm^−2^, which should not be perceived by the patient as painful.

## 4. Materials and Methods

All chemical agents were obtained from POCH (Poland). Preparation of the biogenic gold nanoparticles used in this study was described in Supplementary Information.

### 4.1. Photosensitizer and Light Source

Sodium salt of di- [Al(OH)Pc(SO_3_Na)_2_] and tetrasulfonated [Al(OH)Pc(SO_3_Na)_4_] hydroxyaluminum phthalocyanines were synthesized according to the procedure previously described by Palewska et al. [25]. The phthalocyanine solutions (AlPcS_2_ and AlPcS_4_) were prepared by dissolving the dye in Milli-Q deionized water (UV Ultrapure Water System, Burnstead, USA) and sterilized by filtration using 0.22 µm pore diameter membranes (Millex^®^-HP syringe-driven filter unit, Sigma-Aldrich, Poznan, Poland). These solutions were stored in the dark at room temperature. Diode lasers with the peak-power wavelength λ = 650 nm (output power of 40 mW and 80 mW; radiation intensity of 105 m Wcm^−2^ and 210 m Wcm^−2^) were used in this study.

### 4.2. Dark Toxicity Studies

The reference strain *Staphylococcus aureus* PCM 2054 was cultured in Mueller Hinton broth (BIOCORP, Poland) at 37 °C for 18–20 h. Then, an amount of 2 mL of the culture of *S. aureus* was centrifuged at 1000× *g* for 10 min and washed two times in phosphate buffered saline (PBS). The cell pellet was re-suspended in PBS to give an inoculum of approximately 1.1 × 10^6^ colony-forming units (CFU/mL). The standardized suspension of *S. aureus* was mixed with AlPcS_2_ or AlPcS_4_ solution at final concentrations of 7, 14, and 28 µg/mL and incubated in the dark at 37 °C. After 5, 10, 15, and 30 min of incubation, the percentage reduction (R) of *S. aureus* viability was calculated using the following formula:

R = (N_0_ − N) × 100/N_0_,

where N_0_ and N are the numbers of CFUs in the initial and remaining suspension after incubation with AlPcS_4_ and AlPcS_2_ in the dark. The culture of the studied coccus was incubated under the same conditions and was used as a control (AlPcS_2_ or AlPcS_4_ solution was replaced with PBS). Average and standard deviation of three independent experiments were shown on the graph. The bactericidal activity of the biogenic gold nanoparticles was studied according to the protocol described above for phthalocyanines, with the difference that AlPcS_2_ or AlPcS_4_ solution was replaced with colloidal gold at a final concentration of 20 ppm.

### 4.3. aPDI Studies

The standardized suspension of *S. aureus* was mixed with AlPcS_2_ or AlPcS_4_ solution at final concentrations of 7, 14, and 28 µg/mL and incubated in the dark at 37 °C for 30 min. Then, a 200 µL of each mixture was individually added to well of a 96-well flat-bottom microtiter plate and all these assay groups were exposed to laser light (λ = 650 nm) for 2, 5, 10, and 30 min. Six additional wells containing the bacterial suspension (100 µL) and PBS (instead of phthalocyanine) were prepared. Thee of these samples were exposed to laser light to determine the effect of light alone on bacterial viability, while the remaining three were kept in the dark as an overall control and to determine the initial concentration of bacteria in the suspension. After irradiation, all samples were serially diluted and spread in duplicate onto Mueller–Hinton agar plates. The plates were then incubated aerobically at 37 °C for approximately 24 h. After incubation, the number of viable bacteria was determined and the concentration of survivors expressed as CFU/mL (the obtained results were log transformed). The effect of gold nanoparticles on the effectiveness of photoeradication of *S. aureus* was studied according to the procedure described above. The difference was that instead of the phthalocyanine alone, a mixture of phthalocyanine (7, 14, or 28 µg/mL) and colloidal gold (20 ppm) was used. The detection limit for the number of bacteria was CFUmL^−1^ = 10.

### 4.4. Statistical Analysis

All experiments were run in triplicate and expressed as the arithmetic mean of standard deviation. The results were expressed as the mean of standard deviation. Differences between two means were assessed for significance by the two-tailed Student’s *t*-test. A *p*-value < 0.05 was considered significant.

## Figures and Tables

**Table 1:** The relationship between energy fluence [J cm^−2^]/power density and *S. aureus* cell mortality rate [log_10_ CFU/mL].

Energy Fluence [J cm^−2^]/ Power Density [mW cm^−2^]	*S. aureus*			
	AlPcS_4_	AlPcS_4_ + AuNPs	AlPcS_2_	AlPcS_2_ + AuNPs
	Unit Reduction in Bacterial Cells [log_10_ CFU mL^−1^]			
4.8/105	0.09	3.54	1.32	2.32
12/105	0.88	4.54	2.32	5.42
24/105	3.24	5.14	4.32	*
72/105	*	*	*	*
9.6/210	0.84	4.64	1.42	5.32
24/210	3.2	5.34	4.32	*
48/210	4.54	5.94	*	*
144/210	*	*	*	*

* Lethal effect (the number of bacteria was below the detection limit).

## Data Availability

Not applicable.

## Supplementary Materials

The following are available online, Table S1: The effect of AlPcS2, AlPcS4, and gold nanoparticles (AuNPs) on reduction in viability of *S. aureus* (in dark); Table S2: The effect of laser light alone on the viability of *S.* *aureus.*

## Sample Availability

Samples of the phthalocyanines are available from the authors.
