# Supplementary material for: On the Synergism of Biogenic Gold Nanoparticles and Hydroxyaluminum Phthalocyanines in the Photoeradication of Staphylococcus aureus
Source: Molecules. 2021 Dec 5;26(23):7378. doi: 10.3390/molecules26237378 (PMC8658972; doi:10.3390/molecules26237378)
Supplement: Supplementary file 1 [file molecules-26-07378-s001.zip › molecules-1484840-supplementary.pdf]

On the Synergism of Biogenic Gold Nanoparticles and Hydroxyaluminum Phthalocyanines in the Photoeradication of *Staphylococcus aureus*

Irena Maliszewska

[1] Department of Organic and Medicinal Chemistry, Faculty of Chemistry, Wrocław University of Science and Technology, Wybrzeże Wyspiańskiego 27, 50-370 Wrocław, Poland
* Correspondence: irena.helena.maliszewska@pwr.edu.pl

Supplementary Materials: The following are available online at www.mdpi.com/article/10.3390/molecules26237378/s1,

Synthesis of the biogenic gold nanoparticles. The culture of *C. versicolor* was maintained on PDA, transferred to a fresh agar plate every two months, and grown at 28 °C during 7 days. Then culture was cut into small pieces and transferred to shake-flasks.

The medium consisted of: (per liter): yeast extracts 2 g, KH_2PO4 0.8 g, $MgSO_4x7H_2O$ 0.2 g, $Na_2HPO_4x2H_2O$ 0.25 g, NH_4NO_3 1 g, glucose 10 g; pH 6.0 was used for was used for biomass production. Flasks were incubated at 28 °C for 14 days using orbital shaker at 125 rpm. Then, the culture was centrifuged to separate mycelium. The fresh mycelium was extensively washed with distilled water. The clean mycelium (20 g) was taken into the Erlenmeyer flask, containing 100 mL of Milli-Q deionised water (UV Ultrapure Water System, Burnstead, USA). The gold ions (1 mM of final concentration) was added to the Erlenmeyer flask and agitated at 28^0 C for 48 h (in dark). The control (without the mycelium) was also run along with the experimental flasks. To monitor the formation of the gold nanoparticles the colour of the cell-free filtrate was observed. The biosynthesized gold nanoparticles were easily purified using the sucrose gradient centrifugation technique described previously [1].

1. Maliszewska, I.; Microbial mediated synthesis of gold nanoparticles: preparation, characterization and cytotoxicity studies. Digest J Nano Biostruct, **2013**, *8*, 1123-1131.

Table S1. The effect of $AlPcS_2$, $AlPcS_4$ and gold nanoparticles (AuNPs) on reduction in viability of *S. aureus* (in dark).

Concentration	Values of reduction in viability [%]
$AlPcS_2$ (28 mgL^{-1})	39±1.5
$AlPcS_2$ (14 mgL^{-1})	Below detection limit
$AlPcS_2$ (7 mgL^{-1})	Below detection limit
$AlPcS_4$ (28 mgL^{-1})	22.4±1.5
$AlPcS_4$ (14 mgL^{-1})	Below detection limit
$AlPcS_4$ (7 mgL^{-1})	Below detection limit
AuNPs (20 ppm)	32.4±1.0

Table S2. The effect of laser light alone on the viability of *S. aureus*

**Irradiation time/Energy fluence [Jcm^{-2}]**	**Values of reduction in viability [%]**
2 min/4.8	within the measurement error
5 min/12	within the measurement error
10 min/24	within the measurement error
30 min/72	29±1.0
2 min/9.6	within the measurement error
5 min/24	within the measurement error
10 min/48	15±1.5
30 min/144	44.5±1.0

laser light (λ=650 nm; the radiation power density of 105 mW cm^{-2} and 210 mW cm^{-2})

Accumulation of $AlPcS_2$, $AlPcS_4$, $AlPcS_2$+AuNPs and $AlPcS_4$+AuNPs in *S. aureus* cells. Standardized suspension of *S. aureus* was prepared in PBS (9.5x10^5 colony-forming units, CFU/mL) from the culture grown in Mueller broth. Then $AlPcS_2$ or $AlPcS_4$ at a concentration of 7 μg/mL was added to the bacterial suspension and these samples were incubated in the dark at 37°C for 60 min without shaking.
Subsequently, the cells were centrifuged at room temperature for 5 min at 5000 rpm, re-suspended in PBS, and further incubated in the dark at 37°C for 60 min without shaking. The cells were again centrifuged (5 min, 5000 rpm, room temperature), and absorbance of the supernatant was analysed to determine the amount of the $AlPcS_2$ or $AlPc_4$ transported out of the cells. The absorption measurements were performed on the Shimadzu UV-1650PC spectrophotometer at 630 nm. The similar studies were performed for $AlPcS_2$+AuNPs and $AlPcS_4$+AuNPs mixtures (the biogenic gold nanoparticles was used at a concentration of 20 ppm).
